# Supplementary material for: The search for CDK4/6 inhibitor biomarkers has been hampered by inappropriate proliferation assays
Source: NPJ Breast Cancer. 2024 Mar 4;10:19. doi: 10.1038/s41523-024-00624-8 (PMC10912267; doi:10.1038/s41523-024-00624-8)
Supplement: Supplementary file 6 — Related Manuscript File [file 41523_2024_624_MOESM6_ESM.pdf]

nature portfolio

Corresponding author(s): Adrian Saurin
Reece Foy

Last updated by author(s): Dec 15, 2023

Reporting Summary

Nature Portfolio wishes to improve the reproducibility of the work that we publish. This form provides structure for consistency and transparency in reporting. For further information on Nature Portfolio policies, see our Editorial Policies and the Editorial Policy Checklist.

Statistics

For all statistical analyses, confirm that the following items are present in the figure legend, table legend, main text, or Methods section.

n/a	Confirmed	
☐	☒	The exact sample size (*n*) for each experimental group/condition, given as a discrete number and unit of measurement
☐	☒	A statement on whether measurements were taken from distinct samples or whether the same sample was measured repeatedly
☒	☐	The statistical test(s) used AND whether they are one- or two-sided<br>*Only common tests should be described solely by name; describe more complex techniques in the Methods section.*
☒	☐	A description of all covariates tested
☒	☐	A description of any assumptions or corrections, such as tests of normality and adjustment for multiple comparisons
☐	☒	A full description of the statistical parameters including central tendency (e.g. means) or other basic estimates (e.g. regression coefficient) AND variation (e.g. standard deviation) or associated estimates of uncertainty (e.g. confidence intervals)
☒	☐	For null hypothesis testing, the test statistic (e.g. *F*, *t*, *r*) with confidence intervals, effect sizes, degrees of freedom and *P* value noted<br>*Give P values as exact values whenever suitable.*
☒	☐	For Bayesian analysis, information on the choice of priors and Markov chain Monte Carlo settings
☒	☐	For hierarchical and complex designs, identification of the appropriate level for tests and full reporting of outcomes
☒	☐	Estimates of effect sizes (e.g. Cohen's *d*, Pearson's *r*), indicating how they were calculated

Our web collection on statistics for biologists contains articles on many of the points above.

Software and code

Policy information about availability of computer code

Data collection	No software was used
Data analysis	No software was used

For manuscripts utilizing custom algorithms or software that are central to the research but not yet described in published literature, software must be made available to editors and reviewers. We strongly encourage code deposition in a community repository (e.g. GitHub). See the Nature Portfolio guidelines for submitting code & software for further information.

Data

Policy information about availability of data

All manuscripts must include a data availability statement. This statement should provide the following information, where applicable:

- Accession codes, unique identifiers, or web links for publicly available datasets
- A description of any restrictions on data availability
- For clinical datasets or third party data, please ensure that the statement adheres to our policy

All data supporting the findings of this study are available within the article. Data from individual repeats is in supplementary table 4.

Research involving human participants, their data, or biological material

Policy information about studies with human participants or human data. See also policy information about sex, gender (identity/presentation), and sexual orientation and race, ethnicity and racism.

Reporting on sex and gender	This study used only human cells lines. These were random cells lines, chosen regardless of sex. Total 11 cell lines: 5 male, 6 female.
Reporting on race, ethnicity, or other socially relevant groupings	n/a
Population characteristics	n/a
Recruitment	n/a
Ethics oversight	n/a

Note that full information on the approval of the study protocol must also be provided in the manuscript.

Field-specific reporting

Please select the one below that is the best fit for your research. If you are not sure, read the appropriate sections before making your selection.

☒ Life sciences ☐ Behavioural & social sciences ☐ Ecological, evolutionary & environmental sciences

For a reference copy of the document with all sections, see nature.com/documents/nr-reporting-summary-flat.pdf

Life sciences study design

All studies must disclose on these points even when the disclosure is negative.

Sample size	at least 3 repeats per experiment to account for technical variation, and most experiments contained many biologicasl repeats (often thousands of cells measured)
Data exclusions	No data were excluded from the analyses
Replication	All attempts at replication were successful
Randomization	n/a
Blinding	the investigators were not blinded to treatments.

Reporting for specific materials, systems and methods

We require information from authors about some types of materials, experimental systems and methods used in many studies. Here, indicate whether each material, system or method listed is relevant to your study. If you are not sure if a list item applies to your research, read the appropriate section before selecting a response.

Materials & experimental systems

n/a	Involved in the study	
☐	☒	Antibodies
☐	☒	Eukaryotic cell lines
☒	☐	Palaeontology and archaeology
☒	☐	Animals and other organisms
☒	☐	Clinical data
☒	☐	Dual use research of concern
☒	☐	Plants

Methods

n/a	Involved in the study	
☒	☐	ChIP-seq
☒	☐	Flow cytometry
☒	☐	MRI-based neuroimaging

Antibodies

Antibodies used	rabbit anti-AKT (Cell Signaling Technology, 9272, 1/1000)<br>rabbit anti-pAKT (Ser473)(Cell Signaling Technology, 4060, 1/1000)<br>rabbit-pS6 (Ser235/236)(Cell Signaling Technology, 4856, 1/1000)

	rabbit anti-actin (Sigma, A2066, 1/5000)<br>IRDye 800CW Goat anti-Rabbit IgG (LI-COR, 1/15000)
Validation	Well established antibodes that have been used in thousands of other studies.

Eukaryotic cell lines

Policy information about cell lines and Sex and Gender in Research

Cell line source(s)	hTERT-RPE1 (ATCC, Human Female)<br>MCF7 (ATCC, Human Female)<br>T47D (ATCC, Human Female)<br>NCI-H1299 (ATCC, Human Male)<br>MCF10A (ATCC, Human Female)<br>MDA-MB-231 (ATCC, Human Female)<br>JURKAT (Clone E6-1, ATCC, Human Male)<br>HH (ATCC, Human Male)<br>SKOV3 (CRUK, Human Female)<br>DLD1-FRT (Lab of Stephen Taylor, Human Male)<br>KARPAS-299 (Public Health England, Human Male)
Authentication	All cells were validated by STR profiling
Mycoplasma contamination	All cell lines tested negative for mycoplasma contamination at the start of the study and then routinely screened every 2 months
Commonly misidentified lines<br>(See ICLAC register)	None of the cell lines used in this study are registered as commonly misidentified lines

Plants

Seed stocks	n/a
Novel plant genotypes	n/a
Authentication	n/a
